# Factors Related to Executive Dysfunction after Acute Infarct

**DOI:** 10.1371/journal.pone.0108574

**Published:** 2014-09-23

**Authors:** Ping Hua, Xiao-ping Pan, Rong Hu, Xiao-en Mo, Xin-yuan Shang, Song-ran Yang

**Affiliations:** 1 Sun Yat-sen Memorial Hospital, Sun Yat-sen University, Guangzhou, China; 2 Department of Neurology, Guangzhou First People's Hospital, Guangzhou Medical College, Guangzhou, China; University of Jaén, Spain

## Abstract

**Aim:**

The aim of this study was to investigate the association of infarct location with post-stroke executive dysfunction.

**Methods:**

One hundred seventy-seven patients hospitalized with acute infarction were enrolled. General information and NIHSS score at admission were recorded. The infarct site was recorded from magnetic resonance T2-W1 and FLAIR images, and the extent of white matter disease was assessed using the Fazekas score. Seven days after symptoms, executive function was assessed using the validated Chinese version of Mattis Dementia Rating Scale (MDRS) Initiation/Perseveration (I/P) [MDRS I/P].

**Results:**

The average MDRS I/P score of the 177 infarction patients was 24.16±5.21, considerably lower than the average score (32.7±3.1) of a control group of normal individuals. Patients with infarcts in the corona radiata or basal ganglia had significantly lower MDRS I/P scores that those without infarcts at these locations. The number of infarcts in the basal ganglia was also significantly associated with low MDRS I/P scores. Male gender and low NIHSS score were significantly associated with low MDRS I/P score, and high-density lipoprotein cholesterol was significantly associated with high MDRS I/P score. The number of infarcts in areas other than the basal ganglia as well as corona radiata and the extent of white matter disease had no influence on this score.

**Conclusions:**

The number of infarcts in the basal ganglia corona radiata, low NIHSS score, and male gender are significantly and independently related to poor executive function (that is, low MDRS I/P score) after acute infarct.

## Introduction

Executive function, in tests of cognitive ability, refers to the ability to plan, make decisions, self-correct, and carry out complex activities. As socio-economic development and medical treatment have improved, the quality of life of stroke (infarct) survivors has attracted more and more attention. The incidence of post-infarct cognitive impairment is about 27% to 71% [1], and executive dysfunction is considered to be a core symptom and the first symptom of general cognitive impairment. However, executive dysfunction may also occur alone in some patients [2].

Currently, studies of post-infarct executive dysfunction are few in China, and executive dysfunction is often neglected in clinical practice. No study on factors related to executive function after acute infarction in the Chinese population has yet been published. It is known that early and reliable estimation of functional outcome after acute stroke is important for patients' clinical treatment. To evaluate the relationship of post-stroke executive dysfunction to the location of the infarct, possible risk factors, and white matter disease would be helpful for estimation of functional outcome after acute infarct.

Wen et al. 2004 [3] have suggested that extent of white matter changes (WMC) may affect the type and degree of cognitive impairment in patients with lacunar infarct. They assessed cognition of patients 12 weeks after the lacunar infarct by psychometric tests (Chinese version of Mini-Mental State Examination [MMSE], Alzheimer's Disease Assessment Scale-cognition [ADAS-cog], and Mattis Dementia Rating Scale-Initiation/Perseveration subscale [MDRS I/P]). They used multivariate linear regression analysis and reported that WMC significantly influenced performance in MDRS I/P, but did not independently influence performance on the MMSE and ADAS-cog. Grau-Olivares et al. 2009 [4] have shown that patients with a first-ever infarction present with cognitive impairment (mainly executive dysfunction) in more than half of the cases and that more than 55% of patients fulfill the criterion of mild cognitive impairment of the vascular type.

We have recently investigated risk factors for post-stroke apathy and depression, and found that a low Mattis Dementia Rating Score (MDRS) Initiation/Perseveration (I/P) subscale score was a risk factor [5]. We are now extending the previous findings to investigate the relationship between infarct location and low MDRS I/P score. Therefore, in the present study, we used the validated Chinese version of Mattis Dementia Rating Score (MDRS) Initiation/Perseveration (I/P) subscale [6] to obtain data on the executive function of subjects with acute infarcts and, a week after the infarct, measured executive function and recorded the infarct site, extent of white matter disease, and medical history of patients with acute infarct in order to determine what parameters were associated with an increased incidence of post-infarct executive dysfunction.

## Materials and Methods

This study was approved by the Institutional Review Board of Guangzhou First Municipal People's Hospital (No. 024, 2010). Written informed consent was obtained from all patients before the study. The scale assessments were done by a qualified psychiatric specialist and the testing was confirmed by a psychiatrist and neurologist.

### Patients

One hundred seventy-seven consecutively enrolled patients who had acute stroke from January 2011 to December 2012 were recruited. All strokes were due to acute infarction. The inclusion criteria were the following: patients with a diagnosis of ischemic stroke (IS) using valid ICD-10 codes [7]; stroke occurred within the previous 7 days; computed tomography (CT) was performed to exclude cerebral hemorrhage; acute infarcts with diameter between 2 mm to 2 cm were detected on brain CT or magnetic resonance imaging (MRI) [3]; patients were conscious and their Glasgow Coma Scale (GCS) score was 15; patients cooperated with evaluation scale testing; the NIH Stroke Scale (NIHSS) score was ≤8. The 2 mm to 2 cm size range for the infarcts was chosen to exclude large infarcts and the potential statistical bias that infarct size disparity might cause. But we did not intend to restrict the infarcts to small deep infarcts (such as ‘lacunar’ infarcts) located in the territory of perforating arteries, or infarcts confined to those of small vessel etiology [8].

Patients were excluded based on the following criteria: patients with a diagnosis or case history of atrial fibrillation; a history of schizophrenia, depression, anxiety or other mental illness, including dementia, Parkinson's disease and other neurodegenerative diseases; development of symptoms of severe aphasia so that patients were unable to complete the evaluation; severe heart, liver or kidney dysfunction, malignancies or thyroid disease; contradictions for MRI such as pacemaker implantation and claustrophobia; history of heavy drinking (>42 units per week, where 1 unit equals to 8 gram of alcohol per week) [9]; history of drug abuse or drug dependence; refusal to participate in the study. Patients who were smokers or drinkers were included although heavy drinkers (see above) were excluded. History of smoking was defined as [9] patients who had a history of smoking in the past or were current smokers. History of drinking was defined as [10] patients who had a history of drinking in the past or were current drinkers (<42 units per week). History of cerebrovascular disease was defined as patients who had previously been diagnosed as having had a cerebral infarction or cerebral hemorrhage, according to ICD-10.

### Basic data collection

One hundred seventy-seven patients with acute infarction who met the inclusion criteria were included. Detailed clinical data were recorded including a history of hypertension, diabetes, or history of cerebrovascular disease. The NIHSS score at admission was also recorded. Seven days after infarction onset, the validated Chinese version of MDRS I/P was employed [6]. The MDRS I/P is one subsection of the MDRS. It includes language production and motion coherence, and is mainly used to assess the initiative and sustenance of motion, which are closely related to executive function.

### Image acquisition and preprocessing

MRI scans were acquired using a Siemens Verio 3.0T scanner (Siemens, Erlangen, Germany). We scanned T1-weighted images with the following parameters: repetition time = 1900 ms, echo time = 3.44 ms, inversion time = 900 ms, flip angle = 9°, and voxel size 1×1×1 mm^3^. The FLAIR T2 images were scanned on the axial plane with a slice thickness of 5 mm.

Infarcts in the frontal lobes, temporal lobes, parietal lobe, occipital lobe, basal ganglia, internal capsulem, and corona radiata were recorded according to the T2-W1 together with FLAIR images of the brain magnetic resonance imaging performed after admission. White matter disease was assessed using the Fazekas visual scale score for periventricular white matter and for deep white matter [11], [12].

The T1 images and Flair images for each patient were co-registered linearly in individual space [13]. An experienced radiologist manually drew lesion contours for each patient by referring to the FLAIR T2 images. The volume for each lesion and the total lesion volume for each patient were calculated.

### Statistical analysis

Quantitative data were presented as mean and standard deviation, and categorical data were presented as frequency and percentage. Independent two-sample t-test was performed to compare MDRS I/P scores between patients with and without infarction lesions at specific locations. Univariate regression was used to evaluate the association between patient variables and MDRS I/P score. Multivariate regression was used to identify patient variables that might independently influence performance of MDRS I/P scores. Multicollinearity was evaluated using the variance inflationary factor (VIF), and variables with VIF>5 were considered to have multicollinearity with other covariates and were then excluded from the multivariate analysis. In addition, a healthy control group was used to define executive dysfunction for patient grouping. Healthy controls were elderly and age-matched individuals consecutively enrolled at our hospital for regular annual physical examinations. After ruling out physiological conditions resulting from decline in the level of consciousness or substance use (drugs or medication) as causes of low scores, the presence of executive dysfunction was considered to be a lower MDRS I/P score. Data were analyzed using SPSS 18.0 statistics software (SPSS Inc, Chicago, IL, USA), and a P value<0.05 was considered statistically significant.

## Results

The demography, medical history, laboratory data, and MDRS I/P scores of the study group shown in Table 1. One hundred seventy-seven patients with infarction were included. There were 109 males (61.6%). The average age was 67.4±9.6 years. The average NIHSS score was 2.2±2.5. The average MDRS I/P score was 29.8±6.3. Total lesion volume was 3.1±2.9 cm^3^. Twenty two, 18, 9, 10, 118, 36 and 38 patients were diagnosed with single or multiple infarcts in frontal lobe, temporal lobe, parietal lobe, occipital lobe, basal ganglia, internal capsule, and corona radiata, respectively, (Table 1).

**Table 1:** Summary of patient data (n = 177).

Gender^1^	
Male	109 (61.6)
Female	68 (38.4)
Age (years)^2^	67.4±9.6
Years of education (years)^2^	7.7±4.4
History of cerebrovascular disease^1^	51 (28.8)
History of hypertension^1^	61 (34.5)
History of diabetes^1^	126 (71.2)
Systolic blood pressure (mmHg)^2^	152.7±22.3
Diastolic blood pressure (mmHg)^2^	82.0±14.7
Heart rate (bpm)^2^	75.9±10.3
Total cholesterol (mmol/l)^2^	4.9±1.2
Triglycerides (mmol/l)^2^	1.6±1.2
Low-density lipoprotein cholesterol (mmol/l)^2^	2.9±0.8
High-density lipoprotein cholesterol (mmol/l)^2^	1.1±0.3
Glycated hemoglobin (%)^2^	6.8±2.0
Smoking history^1^	81 (45.8)
Drinking history^1^	49 (27.7)
NIHSS score^2^	2.2±2.5
Fazekas^1^	
Total	2.4±1.5
Periventricular white matter	1.3±0.9
Deep white matter	1.1±0.8
MDRS I/P^2^	29.8±6.3
Number of infarctions in frontal lobe^1^	
0	155 (87.6)
1	13 (7.3)
2 or more	9 (5.1)
Number of infarctions in temporal lobe^1^	
0	159 (89.8)
1	8 (4.5)
2 or more	10 (5.6)
Number of infarctions in parietal lobe^1^	
0	168 (94.9)
1	8 (4.5)
2 or more	1 (0.6)
Number of infarctions in occipital lobe^1^	
0	167 (94.4)
1	5 (2.8)
2 or more	5 (2.8)
Number of infarctions in basal ganglia^1^	
0	59 (33.3)
1	44 (24.9)
2 or more	74 (41.8)
Number of infarctions in internal capsule	
0	141 (79.7)
1	35 (19.8)
2 or more	1 (0.6)
Number of infarctions in corona radiata^1^	
0	139 (78.5)
1	25 (14.1)
2 or more	13 (7.3)

Data are presented as

1 ^1^ n (%);

2 mean ± standard deviation.

Abbreviation: NIHSS, National Institutes of Health Stroke Scale; MDRS I/P, Mattis Dementia Rating Scale (MDRS) Initiation/Perseveration (I/P).

Table 2 compares MDRS I/P score between patients with and without infarcts stratified by location. Patients with infarcts in the basal ganglia and corona radiata had significantly lower MDRS I/P scores than those with no infarcts in these areas (28.7±6.5 vs. 32.0±5.1 in the basal ganglia, P = 0.001; 27.9±7.4 vs. 30.3±5.8 in the corona radiata, P = 0.033). The presence or absence of infarcts in the frontal, temporal, parietal, or occipital lobes did not affect the MDRS I/P score.

**Table 2:** Comparison of MDRS I/P score between patients with and without the infarction lesion stratified by location.

	With	Without	*P* value
Frontal lobe	29.5±5.8	29.8±6.3	0.792
Temporal lobe	28.9±6.9	29.9±6.2	0.549
Parietal lobe	27.4±5.9	29.9±6.3	0.250
Occipital lobe	27.2±6.9	29.9±6.2	0.179
Basal ganglia	28.7±6.5	32.0±5.1	0.001*
Internal capsule	29.0±6.6	30.0±6.2	0.384
Corona radiata	27.9±7.4	30.3±5.8	0.033*

* P<0.05, two independent t-test.

Abbreviation: MDRS I/P, Mattis Dementia Rating Scale (MDRS) Initiation/Perseveration (I/P).

Simple linear regression (Table 3) showed age, years of education, history of cerebrovascular disease, history of hypertension, triglycerides, NIHSS score, Fazekas scores (including total, periventricular, and deep white matter), and number of infarctions in basal ganglia to be significantly correlated with the MDRS I/P score (all, P<0.05). Multiple linear regression showed male gender, NIHSS score, the number of infarctions in the basal ganglia, and the number of infarctions in the corona radiata to have a significant negative association with the MDRS I/P score (all, P<0.05), and high-density lipoprotein cholesterol to have a significant positive correlation with the MDRS I/P score (P = 0.036).

**Table 3:** Simple linear regression and multiple linear regression in the MDRS I/P score.

	Simple linear regression		Multiple linear regression‡	
	β (95% CI)	*P*-value	β (95% CI)	*P*-value
Gender				
Female	reference		reference	
Male	−0.82 (−2.73, 1.09)	0.397	−2.92 (−5.61, −0.23)	0.034*
Age (years)	−0.14 (−0.23, −0.04)	0.006*	−0.13 (−0.26, 0.001)	0.052
Years of education (years)	0.33 (0.12, 0.53)	0.002*	0.15 (−0.09, 0.39)	0.212
History of cerebrovascular disease	−2.51 (−4.53, −0.49)	0.015*	−1.39 (−3.72, 0.94)	0.240
History of hypertension	2.18 (0.25, 4.11)	0.027*	0.64 (−1.58, 2.87)	0.568
History of diabetes	0.11 (−1.94, 2.17)	0.915	0.60 (−2.11, 3.31)	0.661
Systolic blood pressure (mmHg)	−0.03 (−0.08, 0.01)	0.108	0.04 (−0.02, 0.09)	0.214
Diastolic blood pressure (mmHg)	−0.02 (−0.08, 0.05)	0.642	0.04 (−0.05, 0.12)	0.403
Heart rate (bpm)	−0.01 (−0.10, 0.08)	0.803	−0.09 (−0.19, 0.01)	0.088
Total cholesterol (mmol/l)	−0.46 (−1.20, 0.28)	0.223	−1.03 (−2.05, 0.002)	0.051
Triglycerides (mmol/l)	0.82 (0.05, 1.58)	0.037*	0.45 (−0.49, 1.38)	0.345
Low-density lipoprotein cholesterol (mmol/l)	−0.31 (−1.53, 0.90)	0.613	0.56 (−1.19,2.31)	0.525
High-density lipoprotein cholesterol (mmol/l)	0.79 (−2.66, 4.23)	0.653	4.21 (0.28,8.14)	0.036*
Glycated hemoglobin	−0.12 (−0.61, 0.38)	0.647	0.20 (−0.39, 0.79)	0.504
Smoking history	−0.58 (−2.45, 1.28)	0.538	−0.51 (−3.12, 2.10)	0.699
Drinking history	1.37 (−0.70, 3.44)	0.193	1.28 (−1.17, 3.74)	0.302
NIHSS score	−0.83 (−1.22, −0.45)	<0.001*	−0.78 (−1.21, −0.35)	0.001*
Fazekas†				
Total	−0.93 (−1.53, −0.32)	0.003*	−0.21 (−1.00, 0.57)	0.591
Periventricular white matter	−1.36 (−2.41, −0.32)	0.011*	NA	
Deep white matter	−1.76 (−2.94, −0.58)	0.004*	NA	
Number of infarctions				
Frontal lobe	−0.45 (−2.05, −1.16)	0.581	0.17 (−1.42, 1.77)	0.829
Temporal lobe	−0.95 (−2.26, 0.36)	0.155	−1.26 (−3.04, 0.52)	0.164
Parietal lobe	−2.79 (−6.42, 0.85)	0.132	−0.02 (−3.98, 3.94)	0.991
Occipital lobe	−0.98 (−2.74, 0.78)	0.275	−0.59 (−2.70, 1.52)	0.579
Basal ganglia	−1.26 (−2.09, −0.43)	0.003*	−1.40 (−2.43, −0.38)	0.008*
Internal capsule	−0.90 (−3.11, 1.31)	0.424	−1.33 (−3.69, 1.03)	0.266
Corona radiata	−1.44 (−2.89, 0.01)	0.051	−1.75 (−3.45, −0.05)	0.044*

† Fazekas score both in periventricular white matter and in deep white matter were not included in the multivariate analysis due to the significantly collinearity with total Fazekas score.

‡ There is no multicollinearity in the multiple linear regression. Adjusted R^2^ = 0.290.

*P<0.05 indicates a significant correlation.

Abbreviation: CI, confidence interval; NA, not available.

## Discussion

This is the first study to report on factors related to executive function in the Chinese population. In the current study, infarction subjects who had lower MDRS I/P scores (the marker for executive dysfunction used in this study) were more likely to be older, less educated, have a lower NIHSS score, and a higher number of infarctions in the basal ganglia and corona radiata. Multivariate analysis did not show white matter disease or lesions in other brain locations to be independently associated with poor executive function.

The frontostriatal circuitry originating in the orbolateral prefrontal cortex is considered to be the brain region related to executive function. This includes the prefrontal cortex, the basal ganglia, and the fibers in the corona radiata that connect the two regions. A number of studies have reported anatomic evidence of the relationship of this area to executive function.

Alzheimer's patients with low executive function show significant thinning in the dorsolateral and posterior cingulate cortex [14]. Infarction victims with microbleeds in the frontal lobes and basal ganglia also have decreased executive function [15]. Executive dysfunction has been reported to be decreased in infarction patients with infarcts in both cortical and subcortical areas, but not in those with infarcts in one area alone [16]. And executive dysfunction has been reported to be related to infarction in the anterior, but not the posterior brain circulation [17]. However, other studies of infarction victims have found no association between lesion location and executive dysfunction [18], [19].

Our study found executive dysfunction to be related to the number of infarcts in the corona radiata, which is the periventricular region containing fibers that connect the frontal lobe to the basal ganglia. Bombois et al., in a study of patients from a memory clinic who had mild cognitive impairment, found that periventricular white matter intensities (a sign of damage to the corona radiata) were significantly associated with executive dysfunction, but not with memory or other cognitive functions [20]. In our study, although periventricular white matter intensity (as monitored by the Fazekas score) was significantly related to poor executive function in univariate analysis, this relationship lost significance when multivariate analysis was used. Our study found a relationship between poor executive function and number of infarcts in the basal ganglia. But in some studies, a lack of effect of infarcts in subcortical structures on function has been noted. A study on infarcts in human autopsy data found that approximately half of patients with subcortical infarcts had had no clinical symptoms or history of stroke [21]. And two studies in rats in which the effects of subcortical damage were compared to the combined effects of cortical and subcortical damage found that subcortical damage alone had little effect on the functional activities tested (motor performance and rearing activity) [22], [23].

Cerebral microbleeds, due to leakage from abnormal small blood vessels, represent a type of chronic cerebrovascular disease. Werring et al. found infarction victims with microbleeds to be more likely to have executive dysfunction than those without microbleeds. Univariate, but not multivariate, analysis of our data shows that pre-existing cerebrovascular disease is associated with an increased probability of poor executive function [15]. This possibility raises the question, in our study, that poor executive function was present prior to infarction in these patients.

Leonards et al. [24] have shown that severe white matter disease at the time of infarction was associated with disability 1 year later, and Park et al. [25], comparing cerebral microbleeds with white matter damage, have shown that patients with white matter damage are more likely to be older and to have diabetes than those with microbleeds. Mok et al. [26] have further shown that executive dysfunction and left frontal white matter hyperintensities are correlated with neuropsychiatric symptoms in stroke patients with confluent white matter intensities. In our study, univariate, but not multivariate, analysis showed executive dysfunction to be related to the severity of both peripheral and deep white matter damage.

An unanswered question is whether the executive dysfunction observed in our study was partly due to depression. Our previous study [5] showed executive function to be a risk factor for post stroke apathy and depression. Braw et al. [27] have shown that aging is associated with a decline in the frontal lobe-related cognitive functioning of healthy subjects. Depressed patients are impaired in most domains of frontal lobe-related cognitive function. These deficits are already evident at an early age and persist in older age cohorts (despite an overall age related decline) [27]. Bour et al. [28] have reported that symptoms of depression and executive dysfunction are highly prevalent in stroke patients and often co-occur. These patients are more at risk for poor stroke outcome, chronic depression, and cognitive deterioration [27].

Animal experiments, autopsy and neuroimaging studies have shown that after infarction, secondary damage may occur in the subcortical white matter fibers connected with the infarction lesion. After focal cerebral infarction, delayed nerve fiber damage and loss of nerve cells can be detected in some areas far away from the infarct that have a different feeding artery from that of the infarction lesion [24]. It is an unanswered question whether, during the recovery stage, the occurrence of executive dysfunction may be related to the secondary impairment of the white matter fibers connected with the infarct.

This study had some limitations. Because most of the lesions were less than 1 cm in diameter, we could not measure lesion area with enough accuracy to assess the relationship of this parameter to executive dysfunction. Due to the limited number of cases, we did not assess the impact of connected damage in various brain regions on executive dysfunction. We cannot explain why male gender have a significant negative association with the MDRS I/P score and high-density lipoprotein cholesterol have a significant positive correlation with the MDRS I/P score.

In conclusion, the number of lesions located in the basal ganglia and corona radiata, but not the number of lesions in other brain regions, is associated with poor executive function in patients with acute infarcts.
